# Supplementary material for: Effect of a Third Dose of SARS-CoV-2 mRNA BNT162b2 Vaccine on Humoral and Cellular Responses and Serum Anti-HLA Antibodies in Kidney Transplant Recipients
Source: Vaccines (Basel). 2022 Jun 9;10(6):921. doi: 10.3390/vaccines10060921 (PMC9227063; doi:10.3390/vaccines10060921)
Supplement: Supplementary file 1 [file vaccines-10-00921-s001.zip › vaccines-1738233-supplementary.pdf]

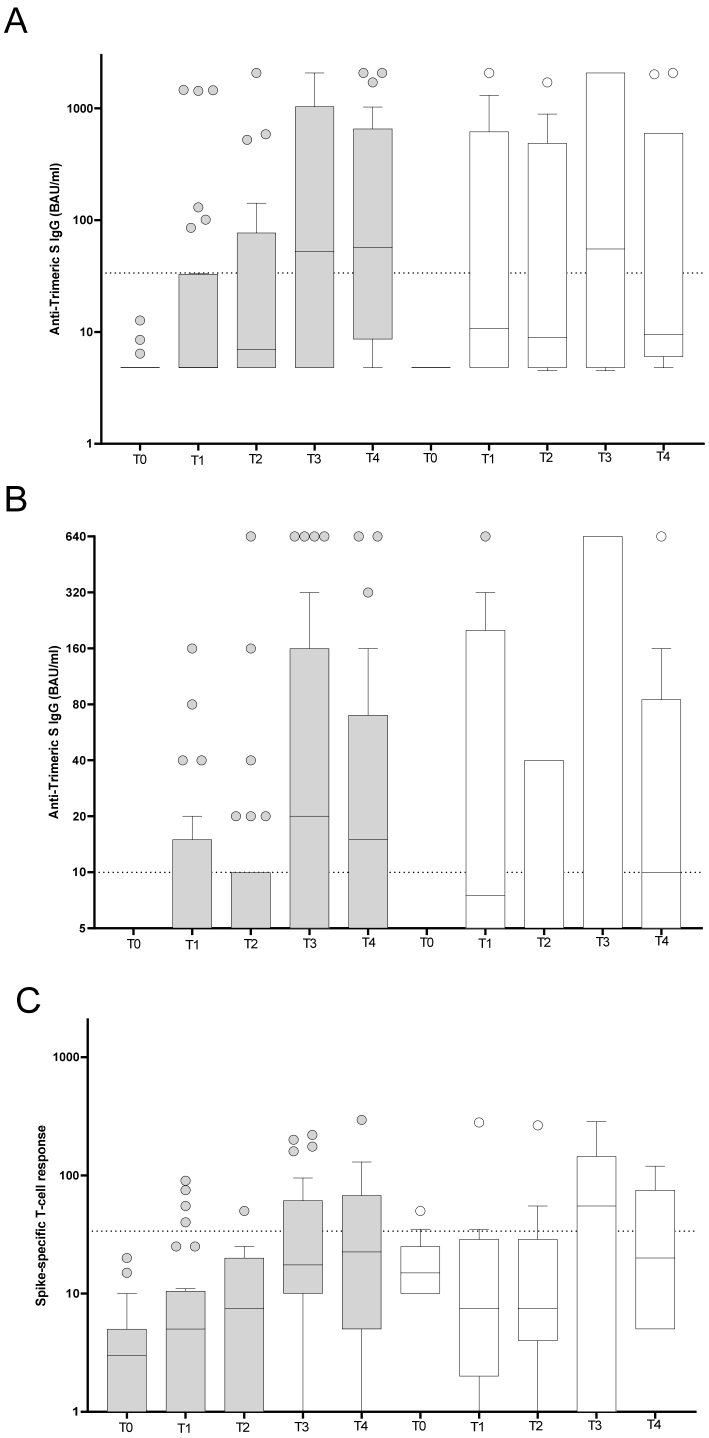


Figure S1. Comparative analysis of immune response elicited by vaccination in Spike-specific T-cell response responders and non-responders at baseline. Subjects with negative Spike-specific T-cell response at baseline (grey boxes) were compared to those with positive Spike-specific T-cell response at baseline (white boxes) for Anti-Trimeric S IgG (A), SARS-CoV-2 NT Abs (B) and Spike-specific T-cell response (C).
